# High-Performance Multilayer Radiative Cooling Films Designed with Flexible Hybrid Optimization Strategy

**DOI:** 10.3390/ma13132885

**Published:** 2020-06-27

**Authors:** Peng You, Xiong Li, Yijia Huang, Xiaoliang Ma, Mingbo Pu, Yinghui Guo, Xiangang Luo

**Affiliations:** 1 State Key Laboratory of Optical Technologies on Nano-Fabrication and Micro-Engineering, Institute of Optics and Electronics, Chinese Academy of Sciences, P.O. Box 350, Chengdu 610209, China; sspeng8808@163.com (P.Y.); qiling006@163.com (X.L.); bradbiubiu@163.com (Y.H.); maxl@ioe.ac.cn (X.M.); pmb@ioe.ac.cn (M.P.); guoyinghui8@163.com (Y.G.); 2 University of Chinese Academy of Sciences, Beijing 100049, China

**Keywords:** radiative cooling, optimization strategy, multilayer design, genetic algorithm, transfer matrix method

## Abstract

Despite their great potential for energy-saving applications, it is still challenging to design passive radiative cooling (RC) materials with simultaneous high performance and simple structures based on traditional design philosophy. To solve the contradiction between optimization speed and corresponding performance, we present a flexible hybrid optimization strategy based on a genetic algorithm (GA) in conjunction with the transfer matrix method and introducing the calculation of radiative cooling power density in the evaluation function of the GA. As a demonstration, an optimized coating with 1.5-μm-overlapping MgF_2_ and Si_3_N_4_ layers on top of a silver film was numerically designed. Based on a detailed analysis of the material’s electromagnetic properties and cooling performance, this coating achieved a radiative cooling power density of 62 W/m^2^ and a temperature reduction of 6.8 °C at an ambient temperature of 300 K. Our optimization strategy may have special significance in the design of high-performance RC materials or other multi-spectral engineering materials with simple structures.

## 1. Introduction

With the acceleration of global warming, the environment is being seriously threatened [1], and thus environmentally friendly cooling methods are highly desired. Passive radiative cooling has attracted much attention in recent years for its properties of considerable cooling performance without energy consumption [2,3,4,5,6]. This technology involves the physical process that objects cool themselves by transferring thermal radiation to the environment through the mid-infrared atmospheric transparency window [5,7]. Nocturnal radiative cooling had been reported in recent decades [8,9,10]. Whereas, daytime radiative cooling shows more advantages for practical applications [3,4,5,11,12,13]. A theory for the design of daytime radiation cooling photonic crystals was proposed by Eden Rephaeli et al. in 2013 [14]; radiative cooling under direct sunlight was first experimentally achieved by Raman et al. in 2014 [2] with a seven-layers film by overlapping SiO_2_ and HfO_2_ layers deposited on a 200-mm silicon wafer. Cooling power density of 40.1 W/m^2^ and temperature reduction of 4.9 °C below ambient temperature was realized with a 2-μm-thick multilayer films. Subsequently, many daytime radiative cooling methods have been proposed, such as polymers [6,15] and metamaterials [5,7]. Although polymers exhibit great potential in radiative cooling according to their visible transparent and infrared emissive characteristics, the degradation of most polymers in outdoor conditions is difficult to restore [16]. On the other hand, it is possible to use metamaterials with subwavelength structures [17,18,19,20,21,22,23,24,25,26] to obtain an optimized radiative cooling. Nevertheless, complex fabrication techniques render mass production difficult.

To achieve effective RC materials, the multilayered film offers significant interest for its ease of processing, thinner thicknesses and flexibility in optimization [2,12,16,27,28]. Traditionally, multilayered RC materials are designed so that their radiative spectra approach an ideal model [11,14,16,28,29]. Typically, the average absorptivity of solar irradiance below 10% and high average mid-infrared emissivity of the materials are often required in the common evaluation principle [14]. However, by selecting the materials and optimizing the layer thickness based on the expertise of the designer, the traditional design is a time-consuming and computational resource-consuming job and often results in locally optimized solutions [30]. Moreover, the fluctuations of solar irradiance and atmospheric transmittance were often ignored in previous designs and the ‘optimized’ materials resulted in a degraded performance.

In this study, we propose a flexible hybrid optimization strategy (FHOS) based on the combination of a GA and the transfer matrix (TM) method to define the number and thickness of layers, simultaneously optimized for an improved radiative cooling power density to solve the general design problem with fast speed and high precision. The problem of interest is to design the RC materials with high performance via optimizing thickness and the order of layers. The GA can search efficiently for optimal solutions including multiple input parameters, which is very suitable for the design of multilayer RC materials. Moreover, the TM method can calculate the electromagnetic properties of the multilayer films with high precision and fast speed. Moreover, the introduction of the cooling power in the evaluation (EV) function leads to a more precise optimization process.

## 2. Materials and Methods

**Figure 1:**
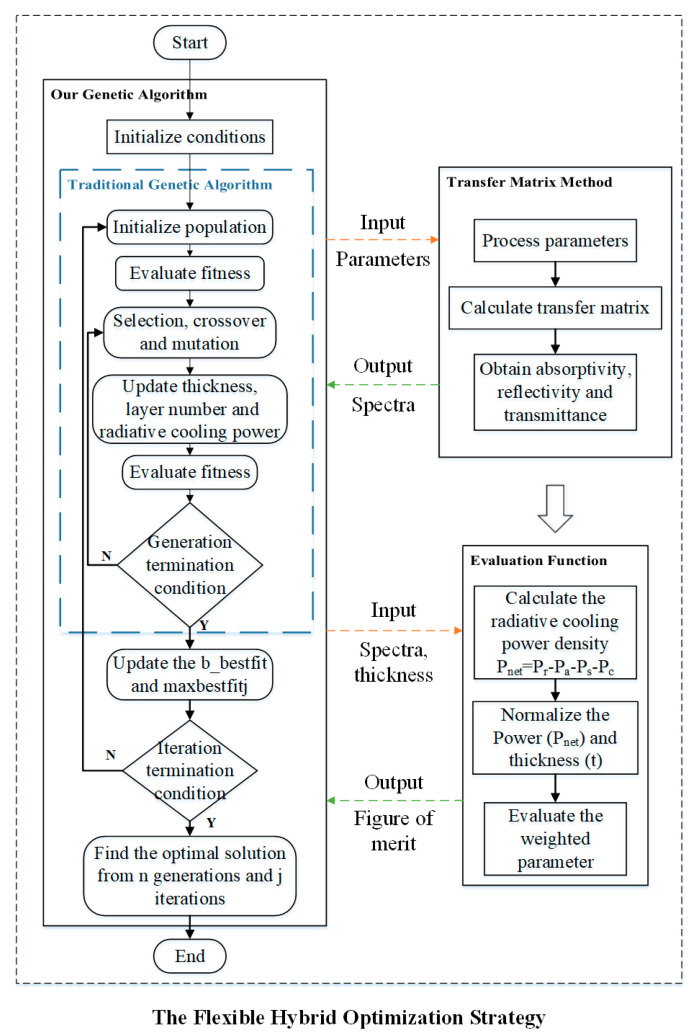
Flow chart of the flexible hybrid optimization strategy (FHOS) for the optimization evaluation. The three parts, the genetic algorithm (GA) (left side), the TM method (upper right) and the evaluation (EV) function (lower right), exchange the information to evaluate the solution candidates.

Our proposed FHOS for the optimization of the multilayered film is composed of three parts exchanging information from each other as shown in Figure 1, the GA (left block in Figure 1), the TM method (upper right in Figure 1) and the EV function (lower right in Figure 1).

In the FHOS, the parameters to be optimized are the layer thickness and layer number. The optimization is managed by the GA, which provides the input values used in the calculation of the electromagnetic (EM) spectra waves by the TM method. The spectra are then input into the EV function for the calculation of the radiative cooling power density and the normalized fitness value. The fitness value is used inside the GA block to sort the solution quality and iterate with the optimization process. The method of the FHOS aforementioned provides an optimized solution to the problem.

The details of each part of the FHOS are described in the following paragraphs.

### 2.1. The GA in the FHOS

The GA always searches in a predefined range of parameters by evaluating a fitness function used as the selection criteria [31,32]. The algorithms require systematic refinement or control tools to avoid converging into a local maximization point instead of the absolute phase space solution.

In the GA—by modularly encapsulating different functions of the algorithm—the stability and flexibility can be improved. In this way, the GA will be more convenient and more stable when combined with other computing models, such as the TM method and the radiation cooling power calculation model applied in this research.

The parameters to be optimized by the GA, layer thickness and layer number, are coded into chromosomes. The chromosomes are divided into many segments, each one representing a gene and corresponding to optimized parameters. Those parameters are initialized as the input of the GA, searching in a predefined range for the optimized solution. Then, after each cycle, the best fitness value of each iteration (b_bestfit) and the maximum fitness value (maxbestfitj) are updated and stored according to the quality of the EV function. To realize the function of optimizing the multilayer material within a certain thickness and a certain number of layers, special processing of the parameters is required for the compilation process of the GA. After the genes are decoded into thickness-related parameters, the thickness of each layer is scaled relative to the maximum thickness. The specific list of layer and thicknesses will input to the TM method for the calculation of the electromagnetic properties of materials with different structures.

### 2.2. The TM Method in the FHOS

For high precision simulation, joint optimization methods based on commercial software simulation are often used together with optimization algorithms, which generally leads to a heavy workload and a long simulation time [33]. Here, for the electromagnetic simulation of multilayered structures, the TM method can work in a faster way [34]. In our optimization strategy, the TM method operates as: [*f*, *R_TE/EM_*, *A_TE/TM_*] = *M*(*H*, *a*, *layer*, *θ_in_*, *n*), the initial parameters input to the TM method are in parentheses, where *H* is the total thickness of the multilayer films, *a* is the ratio of thickness of each layer, *layer* is the number of layers, *θ_in_* is the angle of incidence and *n* is the number of sampling points; the function *M* represents our algorithm of the TM method; what in the square brackets are the output spectra parameters, where *f* is the frequency, *R_TE/TM_* is the reflectivity under TE and TM mode, *A_TE/TM_* is the absorptivity under TE and TM mode. The final spectra are taken as the mean value of these two modes. For example, the final absorptivity (*A*) is calculated with: *A* = (*A*_TE_ + *A*_TM_)/2, where *A*_TE_ and *A*_TM_ is the absorptivity of TE mode and TM mode, respectively.

### 2.3. The EV Function in the FHOS

In our FHOS, the radiative cooling power density is introduced into the EV function of the optimization of RC materials. The effects of the fluctuation of solar irradiance and atmospheric transmittance on cooling performance and the weighting factor of these two parts in the optimization process are precisely considered in this method, which leads to the optimization of radiative cooling material with better cooling performance, but thinner thickness. The theoretical analysis of radiative cooling performance used in our EV function is shown in the following equation. When the temperature of the material is *T*, and the ambient air temperature is *T*_amb_, the net power density (*P*_net_) of the radiative cooling material can be obtained by a rigorous calculation mode [12]:

(1) $$P_{net}=P_{r}(T)-P_{a}(T_{amb})-P_{s}-P_{c}(T_{amb},T)$$

where *P_r_* presents the radiation power density of the coating, *P_a_* is the atmospheric thermal radiation density absorbed by the coating, *P_s_* denotes the absorbed solar power density and *P_c_* is the power loss caused by the thermal convection and conduction between the material and the surroundings. Angular property of the material is appropriately considered in the FHOS for better cooling performance. The spectral and angular emissivity applied in the calculation of the material radiation power (*P_r_*) is expressed as: *ε*(*λ*, *θ*) = *ε*(*λ*)*f*(*θ*), where *ε*(*λ*) is the spectral emissivity of normal direction and *f*(*θ*) presented below is the angular function of the emissivity fitted with calculated spectra of different incident angles.

(2) $$f(\theta )=1-a\times e^{\theta /b}$$

where *a* = 5.5 × 10^−4^ and *b* = 12.05. Above results show that our optimization strategy is suitable for the flexible design of multilayer film structures and achieves a faster optimization speed while effectively avoiding local convergence. Due to the more reasonable evaluation criteria in the optimization strategy, materials with better cooling effect and thinner thickness can be obtained.

## 3. Results

Before the optimization of the structural parameters with specific material category—considering the spectral properties—magnesium fluoride (MgF_2_) and silicon nitride (Si_3_N_4_) were selected as dielectric materials in the optimization strategy. Considering the time of processing and to save the computational resources, a maximum layer number of 10 and a maximum total thickness of 1.5 μm are set in our FHOS. In the calculation, optical constants of the silver film obtained from measured data [35] are adopted in visible and near-infrared wavelength ranges. While the permittivity of silver in mid-infrared wavelength ranges is described by the Drude model $\varepsilon (\omega )=1-\omega_{p}^{2}/(\omega^{2}+i\omega \Gamma )$, where *ω_p_* = 1.13 × 10^16^ rad/s denotes the plasma frequency and Γ = 9.65 × 10^13^ Hz represents the collision frequency [36]. Alternative layers of magnesium fluoride and silicon nitride on a silver film enhance the reflection in visible and near-infrared wavelength ranges and provide high emissivity in the mid-infrared atmospheric transparency window. Optical constants of MgF_2_ [37] and Si_3_N_4_ [38,39] are also obtained from measured data.

**Figure 2:**
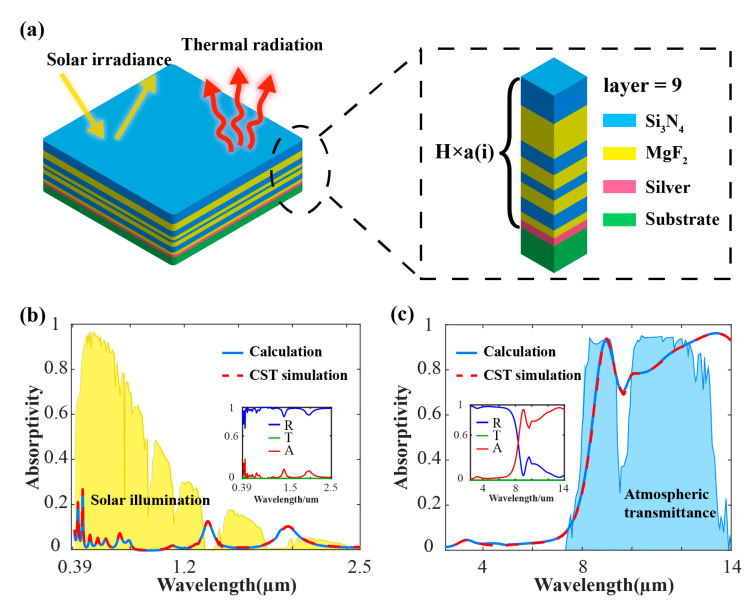
The results of optimized structure. (**a**) Schematic image of the radiative cooling coating with alternating MgF_2_ and Si_3_N_4_ layers; (**b**) calculated and computer simulation technology (CST) simulated absorptivity of the radiative cooling coating over visible and near-infrared wavelength ranges, with the AM1.5 solar spectrum plotted for reference. The coating reflects 95% of the incident solar radiation. The inset is the reflectivity(blue), transmittance (black) and the absorptivity (red) of the coating of 0.39–2.5 μm; (**c**) calculated and CST simulated emissivity/absorptivity of the radiative-cooling coating over mid-infrared wavelengths, with the atmospheric transmittance plotted for reference. The coating emits 76% of the radiation over the mid-infrared atmospheric transparency window. The inset is the reflectivity (blue), transmittance (black) and the absorptivity (red) of the coating of 2.5–14 μm.

After optimization by the FHOS, we numerically presented a radiative cooling coating that maximizing the radiative cooling power density while minimizing the thickness. The optimized coating as shown in Figure 2a was composed of eight alternating transparent dielectric layers, i.e., Si_3_N_4_ and MgF_2_ and a silver reflective film. The total thickness of the coating was 1.5 μm and the specific optimized parameters of the structure are shown in Table 1.

According to the Wein’s displacement law, the central wavelength of the blackbody radiation ranges from 8 μm to 13 μm when the temperature of the object is between 223 K and 362 K. It is well-known from the Kirchhoff’s law that the monochromatic radiation emissivity is equal to the absorptivity when the object is at thermal equilibrium. According to the classical theories and performances of our designed coating, radiation emission to the universe through the mid-infrared atmospheric transparency window can be efficiently achieved with this radiative cooling coating.

The simple structure of the radiative cooling coating brings several benefits. On the one hand, the planar films do not need complex fabrication techniques, such as exposure, erosion or pattern transformation. On the other hand, the spectral responses can be directly calculated by the TM method, which saves much time from the full model simulations. More important, great spectral properties for radiative cooling are demonstrated in this relatively thin coating. The absorption spectra of the radiative cooling coating are calculated (blue solid line) as shown in Figure 2b,c, and the results agree well with the absorptivity calculated by the commercial software CST Microwave Studio (red dashed line). As shown in Figure 2b,c, the absorptivity curves have adaptively adjusted with the fluctuation of the solar irradiance and the atmospheric transmittance. In the visible and near-infrared wavelength ranges where the solar irradiance is extremely concentrated, the temperature rise caused by the solar radiation absorption can be efficiently restrained due to the low absorptivity (high reflectivity) of the coating. In the mid-infrared atmospheric transparency window where the atmospheric transmittance is extremely high, it can be seen in Figure 2c that the coating has also achieved an average absorptivity over 76%.

**Figure 3:**
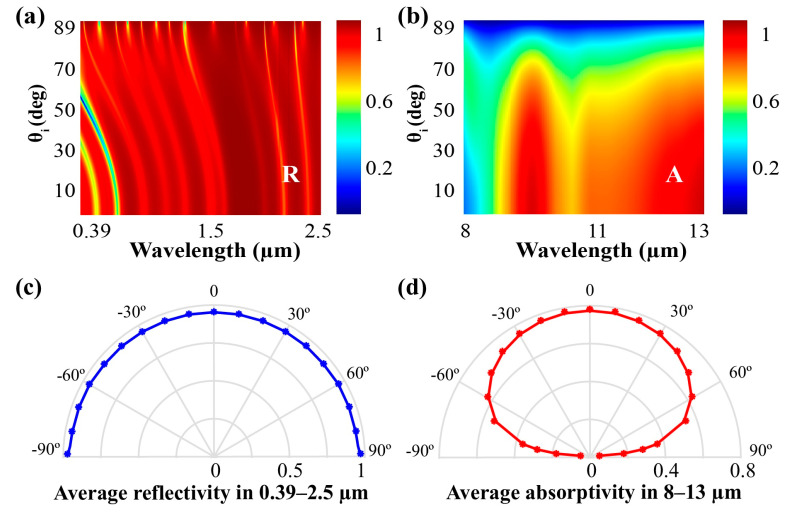
Spectra properties of the radiative cooling material with varying incident angles (θ_i_). (**a**) Reflectivity of the coating of 0.39–2.5 μm with varying incident angles; (**b**) absorptivity of the coating of 8–13 μm with varying incident angles; (**c**) average reflectivity in visible and near-infrared wavelength ranges (0.39–3.5 µm) with the incident angle changes from -90° to 90°; (**d**) average absorptivity over mid-infrared wavelength ranges (8–13 µm) with the incident angle changes from −90° to 90°.

When the radiative cooling coating is exposed to the clear sky, a significant influence on the cooling performance is also presented in the spectral properties for the oblique incidence. The reflectivity in 0.39–2.5 μm and the absorptivity in 8–13 μm under various incident angles are shown in Figure 3a,b, respectively, illustrating the wide-angle characteristics of the designed coating in detail. To further understand of the angular properties of the coating, average reflectivity in 0.39–2.5 μm and average absorptivity in 8–13 μm are separately illustrated in Figure 3c,d with different incident angles. Due to the angular dependent properties of the reflection coefficient introduced in the Fresnel’s equation and the anti-reflection properties of multilayer films, angular insensitivity in 0.39–2.5 μm has been obtained for this coating and the average reflectivity maintains over 0.95 when the incident angle changes from −89° to 89° (Figure 3c). The average absorptivity in mid-infrared wavelength ranges (8–13 μm) as illustrated in Figure 3d is beyond 0.7 under −40° to 40° incident angle, indicating the wide-angle characteristics of this coating in mid-infrared bands.

**Figure 4:**
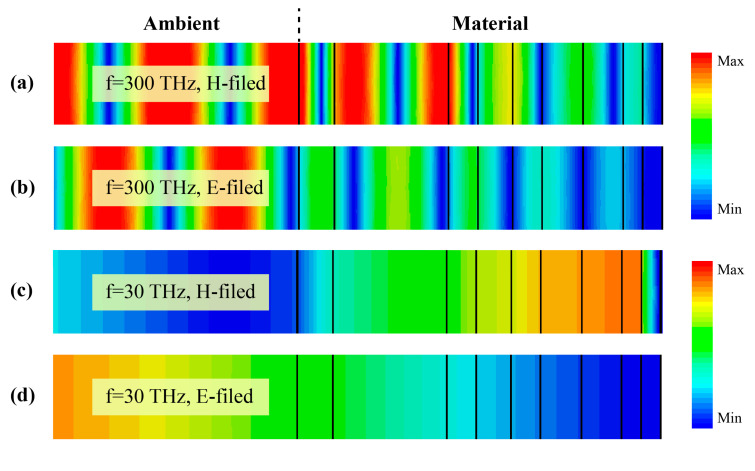
The electric field and magnetic distribution in the simulated material and the ambient. The left side of the dotted line is the domain of the ambient and the right side is the domain of coating. (**a**,**c**) Distribution of the magnetic field at frequency of 300 THz and 30 THz, respectively; (**b**,**d**) distribution of the electric field at frequency of 300 THz and 30 THz, respectively.

For a better understanding of the mechanism of selective reflection in visible and selective absorption in mid-infrared of the coating, the distributions of the electric field and the magnetic field in the materials at two frequencies of 30 THz and 300 THz are provided, respectively. Due to the visible transparency characteristic of MgF_2_ and Si_3_N_4_ [37,39], visible light can pass through these two dielectric materials and almost totally reflected at the silver layer. As can be seen from Figure 4a,b—due to the reflection caused by an impedance mismatch—electromagnetic waves propagate in the form of standing waves in the dielectric material. The increase in the number of layers between the dielectric material of the near-air layer and the metal results in the gradual reduction of the parallel impedance of the dielectric material, which makes the reflection effect closer to the metal layer relatively weaker.

For analysis of the absorption characteristics of the coating of the mid-infrared wavelength ranges, the electric field distribution at a frequency of 30 THz is shown in Figure 4c,d. Since the absorption peak of the Si–N bond is near 11.6 μm [40], silicon nitride has a higher extinction ratio in the mid-infrared band, which in turn produces a strong absorption effect. As a result, the electric field strength gradually becomes weaker in the films. Thus, this coating composed of alternating materials of MgF_2_ and Si_3_N_4_ can exhibit extremely high visible and near-infrared reflectivity and high mid-infrared emissivity.

With the spectral properties of the normal incident and oblique incident of the coating presented above, we numerically analyzed the radiative cooling performance of the coating. The heat coefficient of ambient air is usually set as 6.9 W/(m^2^·K) for calculation [2]. When the ambient temperature is 300 K, this 1.5-μm coating achieves a radiative cooling power density of 62 W/m^2^ under ambient air temperature and a temperature reduction of 6.8 °C below ambient. The total thickness of our designed coating is 1.5 μm, thinner than the existing results [2,12,27,41]. Typically, compared with the previous radiative cooling material designed with multilayered film [2], our coating achieves 130% radiative cooling power with only 75% thickness.

## 4. Discussion

RC materials are often designed by making spectra curve approaches to an ideal band-pass model [11,14,16,28,29]. Minimal average absorptivity of solar irradiance and strongly average mid-infrared emissivity of the materials are often required for radiative cooling under direct sunlight [14]. Whereas the fluctuation of solar irradiance and atmospheric transmittance are often ignored, which result in the optimization of material with a decent spectrum curve, but not the best radiative cooling performance [11,14,16,28,29].

**Figure 5:**
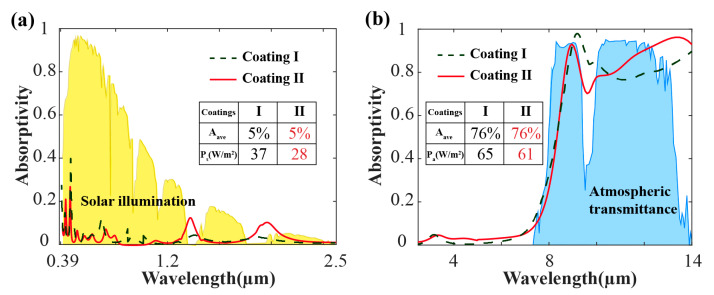
Absorption property of two coatings in the vis-infrared bands and mid-infrared bands. (**a**) Average absorptivity of coating I and coating II in 0.39–2.5 μm is 5%. Absorbed solar irradiance (P_s_) of coating I and coating II are 37 W/m^2^ and 28 W/m^2^, respectively; (**b**) average absorptivity of coating I and coating II in 8–13 μm is 76%. Absorbed atmospheric thermal radiation (P_a_) of coating I and coating II are 65 W/m^2^ and 61 W/m^2^, respectively.

In the design of the radiative cooling films, the radiative cooling performance can be effectively enhanced without increasing the thickness of material. To further verify our point of the adaptive design, we calculated the absorptivity of two coatings in the same way, parameters of these two coatings are given in Table 1. These two coatings have the same total thickness and materials, possessing the equal average absorptivity in 0.39–2.5 μm (5%) and 8–14 μm (76%), respectively. Due to the difference in thickness distribution of different materials, coating I and coating II exhibit different absorption performance. As shown in Figure 5a,b the absorbing curve of coating II (designed with the FHOS) adaptively adjusts with the solar irradiance and the atmospheric transmittance. As a result, coating II absorbs less solar irradiance and less atmospheric radiation while emitting more radiation in the mid-infrared atmospheric window, demonstrated better radiative cooling performance. This phenomenon reveals that by making the spectra adaptively adjust with the fluctuation of the solar irradiance and the atmospheric transmittance, radiative cooling performance is enhanced with our FHOS, compared with the traditional radiative cooling films design.

**Figure 6:**
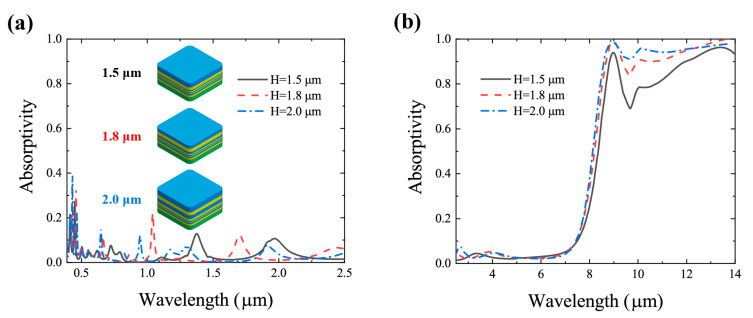
Simulated absorptivity of coatings with same materials composition, but different total thicknesses (H) of (**a**) 0.39–2.5 μm and (**b**) 2.5–14 μm. Coatings with overlapping MgF_2_ and Si_3_N_4_ layers on top of silver film, with total thickness of 1.5 μm (black solid lines), 1.8 μm (red dashed lines) and 2.0 μm (blue dashed lines). The radiative cooling power densities (P) of these three coatings are 62 W/m_2_ for 1.5 μm, radiative cooling power–thickness ratio P/H = 41.3; 69 W/m_2_ for 1.8 μm, P/H = 38.3; and 79 W/m_2_ for 2.0 μm, P/H = 39.5. Materials distribution of these three coatings are the same to the coating demonstrated in Figure 2.

To analyze the influence of the material’s thickness on radiative cooling performance, coatings with overlapping MgF_2_ and Si_3_N_4_ layers on top of silver film are designed with the FHOS, with the total thickness of 1.5 μm, 1.8 μm and 2.0 μm, respectively. As illustrated in Figure 6, with approximately equal visible and near-infrared absorptivity of these three coatings, the thicker coating demonstrates higher absorptivity in mid-infrared bands. However, dividing the radiative cooling power of each coating by its thickness, we can find that the 1.5-μm coating has a higher radiative cooling power–thickness ratio (P/H), which means the better utilization of materials. This result illustrates the rationality of applying the thickness and radiative cooling power density in the EV function of the FHOS.

**Figure 7:**
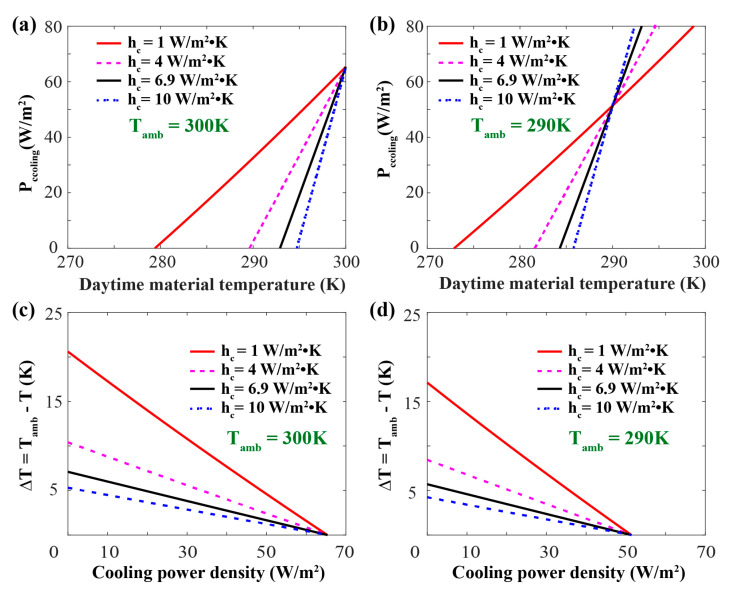
Numerically simulated radiative cooling performance of the structure. (**a**,**b**) Calculated net radiative cooling power density (P_cooling_) of the coating as a function of material temperature with different heat coefficients (h_c_), the ambient air temperatures was set as 300 K in (**a**) and 290 K in (**b**); (**c**,**d**) theoretical calculation for radiative cooling power density as a function of temperature reduction (∆T = T_amb_ – T), the ambient air temperature is set as 300 K in (**c**) and 290 K in (**d**).

The heat coefficient of air is 6.9 W/(m^2^·K) in the above calculation for generalization, which is fitted by experimental data [2]. However, when the radiative cooling material is exposed to diverse environments and situations, the effects of non-radiative heat exchange should be considered. The equilibrium temperature is defined as the temperature when radiative cooling power in Equation (1) is equal to zero and it is also the lowest temperature that can be achieved by the material theoretically [14]. Net radiative cooling power densities of different heat coefficients are calculated with varying temperatures. As illustrated in Figure 7a, the radiative cooling power density is negatively related to the heat coefficient, while the equilibrium temperature is on the contrary, which indicates that the performance of the RC materials can be enhanced with lower non-radiative heat exchange.

The influence of the ambient air temperature is also considered for various conditions. When the material temperature is beyond the ambient air temperature, different from the cases shown in Figure 7a, significant radiative cooling power is obtained with a higher heat coefficient as shown in Figure 7b, which indicates that when the coating is applied for the cooling of higher temperature objects, a larger non-radiation heat coefficient would be more helpful for rapidly cooling. When comparing the equilibrium temperature illustrated in Figure 7a with that in Figure 7b, it can be found that lower equilibrium temperatures can be achieved with lower ambient air temperature, which is mainly caused by the reduction of heat exchange between the designed coating and the surroundings. Besides the equilibrium temperature, the temperature reduction property of the RC materials is often more important in practical applications. As depicted in Figure 7c,d, a similar temperature reduction is achieved with different ambient air temperatures, which indicates that the ambient air temperature has less influence on the cooling function of the RC materials and reveals its wide applications. This discussion helps us to understand the passive radiative cooling in colder weather [42] and design the RC materials with different cooling performance.

## 5. Conclusions

In summary, we have proposed an optimization strategy for the design of high-performance RC materials with simple structures. On account of the introduction of the radiative cooling power density in the EV function and conjunction with the TM method, the FHOS can optimize the RC materials in a faster and more reasonable way. The coating designed with FHOS has demonstrated wide-angle characteristics and desirable cooling performance with a relatively thin thickness, revealing the significance of the FHOS in the increasing integration of RC materials. It is worth noting that the FHOS can also be applied for designing different kinds of other multi-spectral engineering materials, such as multi-band absorbers and high-performance solar cells, by designing the optional functions of the evaluation part. Moreover, ultra-broadband perfect absorber can also be designed with the FHOS if the constraints on the total thickness and layer number are relaxed.

## Figures and Tables

**Table 1:** Optimized parameters of the proposed structure and parameters of coating I and coating II.

Number of Layers			Layer = 9		Total Thickness			H = 1.495 μm	
Layer—i	1	2	3	4	5	6	7	8	9
Material	silver	MgF_2_	Si_3_N_4_	MgF_2_	Si_3_N_4_	MgF_2_	Si_3_N_4_	MgF_2_	Si_3_N_4_
Ratio—a	5.35%	5.55%	11.04%	11.10%	8.16%	9.56%	8.23%	31.24%	9.77%
h-I/nm	80	165	166	122	143	123	467	146	83
h-II /nm	80	83	165	166	122	143	123	467	146
